# The Effect of Spinal Manipulation on the Electrophysiological and Metabolic Properties of the Tibialis Anterior Muscle

**DOI:** 10.3390/healthcare8040548

**Published:** 2020-12-10

**Authors:** Imran Khan Niazi, Ernest Nlandu Kamavuako, Kelly Holt, Taha Al Muhammadee Janjua, Nitika Kumari, Imran Amjad, Heidi Haavik

**Affiliations:** 1 Centre for Chiropractic Research, New Zealand College of Chiropractic, Auckland 1060, New Zealand; kelly.holt@nzchiro.co.nz (K.H.); Nitika.kumari@nzchiro.co.nz (N.K.); imran.amjad@nzchiro.co.nz (I.A.); 2 Faculty of Health & Environmental Sciences, Health & Rehabilitation Research Institute, AUT University, Auckland 0627, New Zealand; 3 Department of Health Science and Technology, Aalborg University, Aalborg 9220, Denmark; taha@hst.aau.dk; 4 Department of Informatics, King’s College London, London WC2R 2LS, UK; ernest.kamavuako@kcl.ac.uk; 5 Faculté de Médecine, Université de Kindu, Kindu, Congo; 6 Faculty of Rehabilitation and Allied Sciences, Riphah International University, Islamabad 46000, Pakistan

**Keywords:** spinal manipulation, maximum voluntary contraction, conduction velocity, near-infrared spectroscopy

## Abstract

There is growing evidence showing that spinal manipulation increases muscle strength in healthy individuals as well as in people with some musculoskeletal and neurological disorders. However, the underlying mechanism by which spinal manipulation changes muscle strength is less clear. This study aimed to assess the effects of a single spinal manipulation session on the electrophysiological and metabolic properties of the tibialis anterior (TA) muscle. Maximum voluntary contractions (MVC) of the ankle dorsiflexors, high-density electromyography (HDsEMG), intramuscular EMG, and near-infrared spectroscopy (NIRS) were recorded from the TA muscle in 25 participants with low level recurring spinal dysfunction using a randomized controlled crossover design. The following outcomes: motor unit discharge rate (MUDR), strength (force at MVC), muscle conduction velocity (CV), relative changes in oxy- and deoxyhemoglobin were assessed pre and post a spinal manipulation intervention and passive movement control. Repeated measures ANOVA was used to assess within and between-group differences. Following the spinal manipulation intervention, there was a significant increase in MVC (*p* = 0.02; avg 18.87 ± 28.35%) and a significant increase in CV in both the isometric steady-state (10% of MVC) contractions (*p* < 0.01; avg 22.11 ± 11.69%) and during the isometric ramp (10% of MVC) contractions (*p* < 0.01; avg 4.52 ± 4.58%) compared to the control intervention. There were no other significant findings. The observed TA strength and CV increase, without changes in MUDR, suggests that the strength changes observed following spinal manipulation are, in part, due to increased recruitment of larger, higher threshold motor units. Further research needs to investigate the longer term and potential functional effects of spinal manipulation in various patients who may benefit from improved muscle function and greater motor unit recruitment.

## 1. Introduction

Chiropractic care is often positioned as a treatment for musculoskeletal pain conditions that is based on improving spinal biomechanics [1,2]. Clinical trials and systematic reviews have shown its usefulness for conditions such as neck pain [3,4,5], back pain [6,7], and some types of headaches [8]. However, over recent years, a growing body of research evidence suggests the beneficial effects of chiropractic care have a neurophysiological basis [9] and these effects extend beyond the treatment of musculoskeletal pain [9,10,11,12,13]. It is important to understand the central neural mechanisms of spinal manipulation, beyond treating pain, as a clearer understanding of the mechanisms may help improve the clinical application of spinal manipulation in other populations. For example, athletes may benefit from spinal manipulation if it can help improve proprioception, strength, and prevention of fatigue. Other populations, such as those who have lost their cortical ability to control their muscles (e.g., stroke survivors), may also benefit from spinal manipulation if it can be shown that spinal manipulation has clinically important implications associated with neuromuscular function.

Multiple basic science studies have shown central plastic changes following spinal manipulation (often referred to as chiropractic adjustments by chiropractors) [14] at the cortical level [9,10,11,12,13,15,16,17,18,19,20,21,22,23,24]. Spinal manipulation has been shown to attenuate cortical (frontal N30) somatosensory evoked potential (SEP) responses [21,22,25]. Most evidence suggests the N30 SEP peak has multiple neural generators and reflects processing within a complex cortical and subcortical loop linking the post-central cortical regions (i.e., S1), the basal ganglia, thalamus, pre-motor areas, and primary motor cortex [26,27,28,29,30,31,32,33,34,35,36,37], and therefore this peak reflects early sensorimotor integration [38]. The specific effects of spinal manipulation on this SEP peak have been shown with dipole source localization techniques to selectively alter prefrontal cortex function [23]. Additional studies have explored whether these SEP changes following spinal manipulation might reflect improved proprioceptive processing following a cervical spinal manipulation. One study was conducted to investigate whether cervical manipulation improved elbow joint position sense [12]. This study did show that manipulating the neck of the participants who had a history of neck dysfunction, but who were not in pain on the day of the experiment, did improve their elbow joint position sense [12]. Another study has shown that chiropractic care for 12 weeks improved ankle joint position sense in older adults [13].

Other motor control changes following spinal manipulation have also been demonstrated [9]. One study has shown that spinal manipulation altered cortical motor control of two upper limb muscles (abductor pollicis brevis and the extensor indicis proprius muscles) in a muscle-specific manner, using transcranial magnetic stimulation (TMS) of the brain [20]. This study used a paired-pulse TMS protocol to explore specific central corticomotor facilitatory and inhibitory neural pathways to the two target muscles. In another TMS study, exploring the input-output characteristics pre and post a single session of chiropractic care, the researchers found that spinal manipulation led to short-term changes in cortical excitability, as measured by a significantly larger maximal motor evoked potential for TMS induced input-output curves for both an upper (abductor pollicis brevis) and lower limb muscle (tibialis anterior muscle), and found larger amplitudes of movement-related cortical potential (MRCP) components [17]. As no changes were found in spinal measures (i.e., F- wave amplitudes or persistence) and no changes were shown following the control condition [17], while changes in the cortical MRCP components, this study suggests that spinal manipulation has a supraspinal neural plastic effect on motor control [17]. Another study also supports this notion, as it showed that spinal manipulation improves tibialis anterior muscle (TA) strength and that this change most likely comes from the supraspinal regions, as only a very small, but significant, change in the H-reflex was observed at low intensities, while large changes were shown in the cortical-based V-wave [15]. Two follow-up studies in elite athletes [10] and chronic stroke patients [11] again showed increased strength of the TA following spinal manipulation, with accompanying large V-wave changes. In both of these studies, no changes in the H-reflex were observed, suggesting spinal manipulation improves the way the supraspinal motor control areas can efficiently produce force.

Combined, these findings suggest that spinal manipulation has an impact on central cortical processing that improves the accuracy with which the brain is aware of limb position and alters the way the brain controls upper and lower limb muscles [9,10,11,12,15]. Several studies have reported increases in muscle strength following spinal manipulation [10,11,15]. Immediate changes in strength following spinal manipulation are likely due to neural adaptations [39,40,41,42]. These may be centrally or peripherally modulated [43]. However, more research is needed to understand the neuromuscular influence of spinal manipulation, because, besides the evidence already discussed, little is known about the neural adaptations that may be associated with increases in strength following a spinal manipulation. These potential neural adaptations can be evaluated by assessing aspects of motor unit behavior, such as changes in motor unit discharge rate, motor unit recruitment, and changes in the velocity of propagation of motor unit action potentials across the muscle fibers [39,44,45]. These neurophysiological aspects of motor unit behavior can be characterized using high-density surface electromyography (HDsEMG) or intramuscular electromyography (iEMG) [46,47].

In addition to gaining a better understanding of the neurophysiological characteristics of motor unit behavior following spinal manipulation, it is also important to better understand whether spinal manipulation has an impact on metabolic factors that may alter motor control. Especially, the balance between oxygen supply and its consumption during muscular activity is very important as it influences adenosine triphosphate (ATP), which is critical for the contractile activity of skeletal muscles [48]. To better understand whether spinal manipulation has an impact on metabolic factors that may influence motor control, near-infrared spectroscopy (NIRS) may be used to monitor regional tissue oxygenation, hemodynamics, and metabolism in skeletal muscles [49,50,51]. This may be very important when evaluating whether any changes in neuromuscular fatigue following spinal manipulation result from central and/or peripheral mechanisms [52,53]. Therefore, to further explore the motor control changes that are known to occur to the TA muscle after spinal manipulation, this study aimed to investigate the electrophysiological and metabolic properties of the TA muscle before and after a single session of spinal manipulation using NIRS and HDsEMG.

## 2. Methods

### 2.1. Design and Setting

This study was a randomized controlled crossover trial with a minimum seven-day washout period between study sessions. The study was a collaborative study conducted in two separate laboratories, Aalborg University, Aalborg, Denmark, and the New Zealand College of Chiropractic in Auckland, New Zealand. The data on electrophysiological measures were collected from Aalborg University, while the metabolic measures were collected at the New Zealand College of Chiropractic. This was done because of the availability of equipment. The same research investigator undertook all the measurements from the two laboratories to ensure there was no bias due to difference in measurement skills of research investigators. This study was conducted following the Declaration of Helsinki and was approved by the local ethical committee of the North Jutland Region (approval no: N-20140027) and the Northern A Health and Disability Ethics Committee, Auckland (approval no: 16NTA9). All participants gave written informed consent before participating in the study.

### 2.2. Participants

Volunteers were eligible to be included in this study if they were English speaking, aged 18–50 years, and had some history of recurring spinal dysfunction, such as mild pain, ache, and/or stiffness with or without a history of known trauma (subclinical pain). Volunteers were ineligible to participate if they exhibited no evidence of vertebral subluxations (biomechanical lesions of the spine that cause maladaptive neural plastic changes) [11], had absolute contraindications to spinal manipulation (including spinal fracture, atlantoaxial instability, spinal infection, spinal tumor, or cauda equina syndrome), had experienced a previous significant adverse reaction to spinal manipulation (defined as an untoward occurrence that is life-threatening, requires hospital admission, or results in significant or permanent disability) [54], they were suffering from a current lower limb disorder/dysfunction that would make them unable to carry out data recording sessions (e.g., sprain/strain/fracture), or if they had sought treatment for their subclinical pain symptoms. Participants were identified via advertising at the New Zealand College of Chiropractic and Aalborg University.

Twelve participants were recruited for the electromyography (EMG) assessment in Denmark (age range 25–33 years, mean 28.3 ± 2.6 years, 8 male, 4 female) and 13 participants were recruited for the near-infrared spectroscopy (NIRS) assessment in New Zealand (age range 23–37 years, mean 29.1 ± 4.6 years, 7 male, 6 female). Overall, 25 participants were recruited across the two settings (age range 23–37 years, mean 28.7 ± 3.66 years, 15 male, 10 female).

### 2.3. Sample Size

Sample size calculations were based on detecting a difference in a continuous response variable from independent control and experimental sessions. Calculations were made based on a previous study that investigated changes in the force of lower limb muscles pre and post a spinal manipulation session [15]. If the true difference between the experimental session and the control session had an effect size of 0.5, we needed 11 participants to be able to reject the null hypothesis that the population means of the experimental and control sessions were equal with probability (power) 0.8. The type I error probability associated with the test of this null hypothesis was 0.05. To allow for attrition during the trial and the relative uncertainty relating to power outcomes, we aimed to enroll 13 participants in each aspect of the trial (EMG and NIRS assessments), which meant a target of 26 participants for the overall trial.

### 2.4. Randomization and Blinding

The allocation of participants was carried out using an online randomization program. The randomization sequence was created using QMinim (Telethon Kids Institute, Perth, Australia) with a 1:1 allocation to receive the control or experimental intervention first. The allocation was performed after the baseline assessment had taken place. Participants and the chiropractors providing care during the study were not blinded to group allocation. All participants were informed that they would receive spinal manipulation or a series of passive movements depending upon whether they were assigned to the intervention or control group on the day of assessment. Outcomes assessors and data analysts remained blinded to group allocation throughout the study period.

### 2.5. Experimental Procedure

Following the eligibility assessment and informed consent procedures, participants underwent a baseline evaluation prior to group allocation. They then received the appropriate intervention before being reassessed immediately post-intervention. Participants were reassessed using the same procedure, but with the alternate intervention, following a minimum seven-day washout period.

### 2.6. Interventions

The study involved two interventions: a single session of spinal manipulation or passive movement control.

#### 2.6.1. Spinal Manipulation

The entire spine and both sacroiliac joints were assessed for vertebral subluxations and manipulated using high-velocity, low-amplitude spinal manipulation when necessary. The clinical indicators that were used to assess for vertebral subluxations included assessing for tenderness to palpation of the relevant joints, manually palpating for a restricted intersegmental range of motion, assessing for palpable asymmetric intervertebral muscle tension, and any abnormal or blocked joint play and end-feel of the joints. These indicators are reliable for the identification of vertebral subluxations when used as a multidimensional battery of tests [55].

#### 2.6.2. Control Intervention

The control intervention involved a series of passive movements that mimicked the movements that were performed in the chiropractic intervention, except no manipulative thrusts were applied. This control intervention was primarily intended to act as a physiological control for possible changes occurring due to the cutaneous, muscular, or vestibular input that occurred with the movements involved in the chiropractic intervention session.

### 2.7. Outcome Measures

Outcome measures were assessed immediately pre and post the two different interventions. The outcome measures that were assessed were maximum voluntary contractions (MVC’s) of the ankle dorsiflexors, conduction velocity across the TA (using HDsEMG), TA motor unit discharge rate (using intramuscular EMG), and TA oxygen consumption (using NIRS). It was not possible to conduct these NIRS and EMG assessments concurrently in the same people due to the devices used and the experimental set-up and protocols that were required. Therefore, MVC’s were assessed in all participants, but half of the participants underwent the EMG assessment (in the laboratory at Aalborg University in Denmark) and the other half underwent the NIRS assessment (in the laboratory at the New Zealand College of Chiropractic in New Zealand).

#### 2.7.1. Intramuscular and High-Density EMG Experimental Procedure (Including MVC’s)

Participants were seated comfortably in a chair. An HDsEMG electrode array with an inter-electrode distance of 8 mm (ELSCH064R3S, OT Bioelectronica, Torino, Italy) was vertically strapped on to the TA muscle. Before attaching the electrode grid, the skin was cleaned with alcohol. The HDsEMG was sampled at 2048 Hz with a gain of 2000 (OT Bioelectronica, Torino, Italy). iEMG signals were recorded to measure motor unit discharge rates pre and post the interventions. A single pair of wire electrodes were inserted in the muscle to record iEMG signals. The location was between the most distal innervation zone, identified as previously described [56], and the distal tendon. Intramuscular wire electrodes were made of Teflon-coated stainless steel (50 µm diameter, A-M Systems, Sequim, WA, USA) and were inserted into each muscle with a sterilized 25-gauge hypodermic needle. The insulated wires were cut to expose 3 mm of wire from the tip [57]. The needle was inserted to a depth of approximately 10–15 mm below the muscle fascia and then removed to leave the wire electrodes inside the muscle. The iEMG was sampled with 10 kHz with a gain of 1000 (OT Bioelectronica, Torino, Italy).

**Figure 1:**
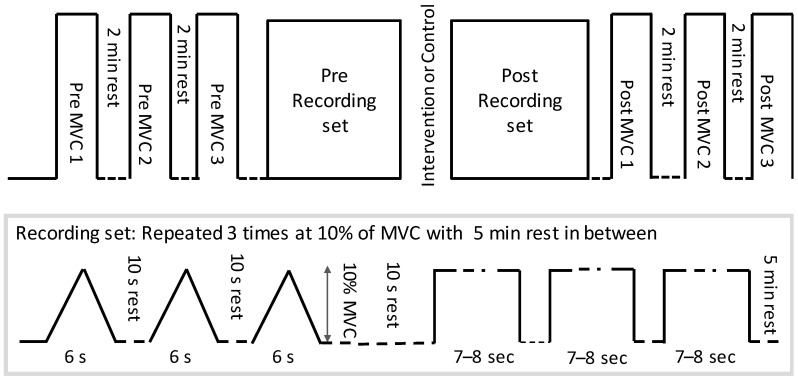
The protocol followed by each participant. MVC = maximum voluntary contractions.

After the injection of the wire electrode and attachment of HDsEMG electrodes, the right leg was fixed to a custom-made pedal for ankle joint torque measurements. Participants performed three ankle dorsiflexion MVC’s and the highest value was retained to compute 10% MVC and potential change in pre/post MVC. After the MVC was determined, the participants performed three repetitions of 7–8 s steady-state isometric dorsiflexion and ramp contractions (ramping up for 3 s and down for 3 s) at 10% of MVC (See Figure 1). Ten percent of MVC was chosen to specifically investigate the low threshold single motor units, because only the threshold of the H-reflex (i.e., reflecting low threshold motor units) was shown to change following a spinal manipulation intervention by Niazi et al. 2015 [15]. The order of isometric and ramp contraction was randomized. These measurements were performed before and immediately after the intervention (chiropractic vs. control) while keeping the same 10% force target as initially computed based on the baseline MVC.

#### 2.7.2. Near-Infrared Spectroscopy Experimental Procedure (Including MVC’s)

Near-infrared spectroscopy measurements were obtained with a continuous wave system (Oxymon MK III; Artinis Medical Systems, PW Elst, The Netherlands) using two wavelengths (850 and 760 nm). A single channel composed of one transmitter optode and one receiver optode was placed over the mid-belly of the TA muscle to measure the relative changes in oxygenated hemoglobin (O_2_Hb).

The NIRS assessment was conducted with the participants seated comfortably in a chair. After placement of the NIRS optodes, the right leg was fixed to a custom-made pedal for ankle joint torque measurements. Subjects performed three ankle dorsiflexion MVC’s and the highest value was retained to compute 30% MVC and the potential change in pre/post MVC. Thirty percent was chosen to allow significant oxygen consumption in the muscle. After the MVC was determined, subjects performed three repetitions of 30 s steady-state isometric dorsiflexion contractions at 30% MVC. These measurements were performed before and immediately after the intervention (chiropractic vs. control) while keeping the same 30% force target as initially computed before the intervention.

### 2.8. Data Analysis

#### 2.8.1. Conduction Velocity with High-Density EMG

HDsEMG was used to calculate the conduction velocity across the TA using algorithms of cross-correlation and a maximum likelihood multi-channel approach adapted from Farina et al. (2004) [58]. This method involved iteratively cross-correlating signals in a multi-channel matrix to find the electrodes whose signals had the least mean square error while calculating the similarity between them.

#### 2.8.2. Intramuscular EMG

iEMG was digitally bandpass filtered between 100–3000 HZ using a 2nd order zero-phase shift Butterworth filter. The signals were fully decomposed using EMGLAB [59] into constituent motor unit action potentials (MUAPs). Following MUAP decomposition, the average MUAP discharge rate (ADR) was computed for the three seconds in the middle of the contraction. Subsequently, the mean force was calculated within the same interval.

#### 2.8.3. NIRS Signals

NIRS signals (O_2_Hb) were pre-processed using a moving average filter of one second in duration and then corrected for blood volume as previously proposed [60]. For each signal modality, the first five samples were averaged and then subtracted from the signal to remove any offset and bring the starting point to zero. Thereafter, the minimum value (mO_2_Hb) was computed as an indication of maximum oxygen consumption during the contraction.

### 2.9. Statistical Analysis

The MVC data were pooled together from both the EMG and NIRS groups. A two-way repeated-measures analysis of variance (ANOVA) was used to assess for differences between the effects of a single session of spinal manipulation or the passive movement control, on force with time (pre and post) and intervention (spinal manipulation and passive movement control) as factors. Similarly, a two-way repeated-measures ANOVA was used with conditions (ramp and isometric) and interventions (spinal manipulation and passive movement control) to assess the percentage change in conduction velocity. For ADR and mO_2_Hb, one-way repeated measures ANOVA’s were used to assess for differences in percentage change (from pre- and post-intervention measures) between interventions (spinal manipulation and passive movement control). Post hoc pairwise comparisons were assessed using Tukey’s HSD tests, and an alpha level of 0.05 was used to determine statistical significance for all tests.

## 3. Results

### 3.1. MVC

There was a significant group * time interaction for MVC’s F (1.24) = 10.5; *p* < 0.01. Overall, there was an increase in MVC of 18.87 ± 28.35% (*p* = 0.02) following the spinal manipulation session and a non-significant decrease (*p* = 0.12) in MVC of −8.14 ± 17.53% following the control session. There was no significant difference in baseline values between interventions (*p* = 0.09).

### 3.2. Discharge Rate/Firing Frequency (Intramuscular EMG)

**Figure 2:**
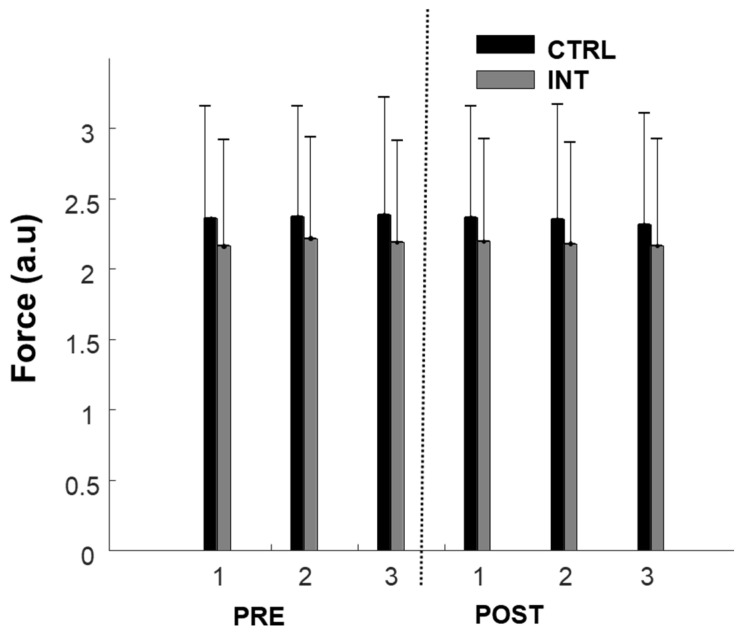
Force production during 10% maximum voluntary contractions (MVCs) pre- and post-intervention for the control group (black) and intervention group (grey). 1, 2, and 3 represent repetitions of contraction consecutively.

Participants were able to produce consistent force levels (10% MVC) before and after the intervention in both groups as depicted in Figure 2. For the ADR, no difference was found between the three repetitions of each block (pre- or post-intervention), suggesting consistency in the experimental conditions. Thus, the three repetitions were averaged for each feature before pre and post comparisons. There was a non-significant decrease in ADR in both the control group (−2.2 ± 4.7%, F = 0.985; *p* = 0.3) and experimental group (−12.5 ± 6.3%, F = 2.86; *p* = 0.1).

### 3.3. Conduction Velocity (HDsEMG)

**Figure 3:**
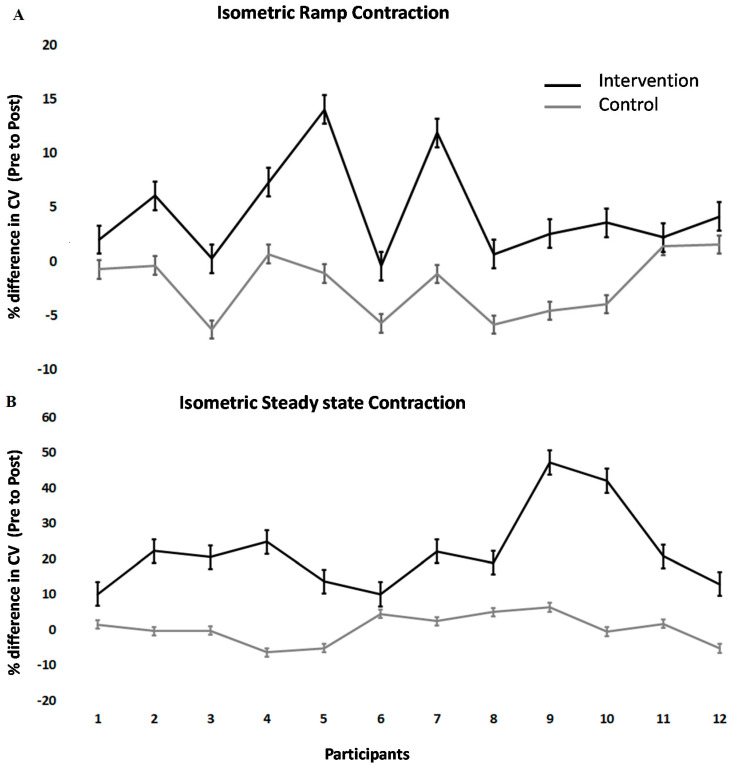
Percentage change post- versus pre-intervention in conduction velocity at 10% MVC for (**A**) isometric steady-state, (**B**) isometric ramp contractions. The error bar is given as the standard error of the mean.

Conduction velocity changes across TA differed between interventions (F (1.11) = 21.2; *p* < 0.001). Following the spinal manipulation intervention, conduction velocity in the isometric steady state increased significantly by 22.11 ± 11.69% (*p* < 0.001). Following the control intervention, it remained unchanged (+0.28 ± 4.17%). During isometric ramp contractions, the conduction velocity also significantly increased by 4.52 ± 4.58% (*p* < 0.001) in the intervention group but remained unchanged following the control intervention (+2.19 ± 2.94%). The values of conduction velocity varied from 4 to 2 m/s, which was expected, considering previous studies showed similar results [58,61]. Individual changes can be seen in Figure 3.

### 3.4. Metabolic Properties

No difference in mO_2_Hb was found between the three repetitions of each block (pre- or post-intervention), suggesting consistency in the experimental conditions. The three repetitions were averaged before pre and post comparisons. Following the chiropractic intervention, there was a −13 ± 9% reduction in the O_2_Hb concentration, whereas the control group change was 2 ± 10%. However, no difference was found between the two groups (F (1.12) = 1.47; *p* < 0.2).

## 4. Discussion

This study aimed to assess the effect of a single session of spinal manipulation on the electrophysiological and metabolic properties of the TA muscle. Results showed in both experiments that, after a single spinal manipulation session, the MVC force increased significantly, suggesting that motor control was altered. The results also revealed that after the chiropractic intervention, the average conduction velocity of the TA increased significantly both in the isometric steady-state and during the isometric ramp contractions compared to the control session. There were no other significant findings.

### 4.1. Chiropractic Adjustments Alter Muscle Strength

Multiple previous studies have documented strength increases following a spinal manipulation. One study demonstrated a 16% increase in plantar flexor muscle strength after a single session of spinal manipulation in healthy participants [15]. Another study in elite taekwondo athletes found that even in this group of athletes, there was an 8% increase in plantar flexor muscle strength following a single session of spinal manipulation [10]. Another study, in subjects with patellofemoral pain syndrome (PFPS), found that a single session of chiropractic care increased quadriceps strength [62]. This finding was similar to a previous study, which showed an increase in quadriceps strength following spinal manipulation in participants with anterior knee pain [63]. An increase in muscle strength following spinal manipulation has also been noted in other muscle groups, such as the upper limb, trunk, and jaw muscles [64,65,66,67]. Interestingly, a significant increase in bite force, which was retained up to one week after the spinal manipulation session, was noted in people with a history of mild recurring spinal dysfunction, indicating maintenance of the beneficial effects of spinal manipulation [64]. Although most of these studies have been conducted in relatively healthy populations, a recent study was conducted in a group that had lost their ability to cortically activate their muscles, i.e., chronic stroke patients with ongoing plantar flexor muscle weakness [11]. Despite that, these chronic stroke patients, with ongoing lower limb muscle weakness, showed a significant increase in plantarflexion muscle strength by on average 64.2% following a single session of spinal manipulation [11]. The greater percentage increase in strength in this stroke study compared to previous studies may be due to the stroke patients having weaker muscles to begin with, so they had more opportunity to increase in strength. The current study finding that both groups increased plantarflexion strength after the spinal manipulation session is congruent with these previous findings. The current study results also help to elucidate the mechanisms of how spinal manipulation can impact human motor control.

### 4.2. Electrophysiological and Metabolic Changes

The current study adds insight into mechanisms associated with strength increases that occur after spinal manipulation. There are three main ways human muscles can increase in strength; (a) an increase in discharge rate of pools of motor units, (b) recruitment of more motor units or (c) changes in the contractile apparatus itself [68,69,70]. As spinal manipulation is performed in less than a few minutes, it is unlikely this alters the contractile apparatus of muscles; thus, strength increases after a manipulation session are more likely due to a change in either recruitment of motor units or their discharge rate. The novel aspect of this study is that the results suggest the strength changes observed following spinal manipulation are, at least in part, due to the recruitment of new motor units, as we found an increase in the average TA conduction velocity after spinal manipulation. Previous studies have shown that the conduction velocities of action potentials along muscle fibers are associated with fiber diameter [71,72]. While every single participant increased their conduction velocity after the spinal manipulation intervention, it is interesting to note the individual differences. Some participants clearly increased their conduction velocity more than others. After the control intervention on the other hand, all the participants either decreased their conduction velocity slightly or remained unchanged. It would be interesting in future studies to explore these individual differences and what they may mean clinically for each person.

No change in the discharge rate was found in the current study. This further supports the above hypothesis about the recruitment of new units. Despite the increase in the selectivity of the used electrode (3 mm exposed), the sensing area was still limited. Nonetheless, it is also possible that spinal manipulation did not cause changes to the motor unit discharge rate. One previous study, measuring single motor unit discharge rates in TA (at very low contraction levels), also reported no change in single motor unit discharge rates following spinal manipulation [18]. This same study did, however, show that the spinal manipulation intervention reduced the threshold (increased the excitability) of low-threshold motor units [18] thus supporting the current finding of changes in the recruitment of motor units without a change in the discharge rate of these motor units.

There was no change in the metabolic activity of the TA after spinal manipulation in the current study. Thus, as the same force level was applied, the needed oxygen supply remained the same and was not altered by spinal manipulation. It is possible that spinal manipulation affects primarily larger fibers and therefore, this effect cannot be revealed at low submaximal levels (<40% MVC) as used in this study. One previous study, using TMS-induced stimulus-response curves, found that for an upper limb muscle, there was only a significant difference in outputs at the higher contraction levels [17]. However, in the same study, for the lower limb TA muscle, they found a shift of the entire input-output curve after the spinal manipulation session [17].

This change in motor unit recruitment following spinal manipulation is likely due to changes originating at a supra-spinal level. Several transcranial magnetic stimulation (TMS) studies have shown that spinal adjustments alter the stimulus-response curve characteristics of both an upper and lower limb muscle [17] and impact various intracortical facilitatory and inhibitory networks [20], without changes in F wave parameters [17,20]. The F wave reflects the antidromic activation of a portion of the lower motor neurons at the spinal cord level [73], so reflects spinal cord excitability. Several studies have also shown that there is either no change or minimal change in the H-reflex following spinal manipulation, despite the significant increases in strength that have been observed [10,11,15]. The H-reflex reflects the excitability of the synapse between large, fast-conducting Ia fibers and lower motor neurons [73] and is largely modulated by presynaptic inhibition and lower motoneuron excitability [74] and therefore also reflects spinal level excitability. However, multiple studies have reported a relatively large significant increase in the volitional (V) waves/voluntary activation that accompany the strength increases after spinal manipulation [10,11,15]. The V-wave is a measure of supraspinal input, or cortical drive, to the motor neuron pool [75,76]. This increase in V-wave amplitude, combined with no significant changes in H-reflex parameters, indicates that the increased strength following spinal manipulation is most likely modulated, to a large degree, at a supraspinal level.

The change in muscle strength found in the current study may be associated with alterations in somatosensory processing at the cortical level. The prefrontal cortex is one possible candidate for the origin of these changes in supraspinal motor control observed following spinal manipulation, as another study has shown, using dipole source localization, that the neural generator of the N30 somatosensory evoked potential (SEP) peak amplitude, that has consistently reduced in amplitude in several studies following spinal manipulation [22,25] was occurring within the prefrontal cortex [23]. Thus, spinal manipulation appears to alter somatosensory processing at the cortical level, particularly within the prefrontal cortex [23]. This, in turn, may impact motor unit recruitment and thus increase muscle strength. The prefrontal cortex has been shown to be involved in the improved brain function found following exercise in older subjects [77]. Even in middle-aged people, moderate-to-high levels of physical exercise benefit the planning and execution of motor response and the executive functions mediated by the PFC [77]. To measure the planning and execution of the motor response, these authors recorded MRCPs, and the changes in these MRCPs, post-exercise, were source localized to be occurring in the prefrontal cortex [77]. Interestingly, MRCP amplitudes have also been shown to increase following spinal manipulation [17]. It is, therefore, possible that this increase in MRCP amplitude, which has been shown to occur following spinal manipulation [17], also reflects changes in movement planning and execution that are occurring at the level of the prefrontal cortex that is influencing motor unit recruitment and strength. This, in turn, would increase the conduction velocity of action potentials along the fibers of the muscle, as was seen in the TA muscle after spinal manipulation in the current study.

### 4.3. Limitations of the Study

This study performed experiments in two separate laboratories with separate groups of subjects, and thus the link between electrophysiological changes and metabolic changes cannot be established with certainty, even though the maximum force increased in both groups after spinal manipulation. Further studies should make concurrent measurements, especially with currently available miniaturized EMG grids and wireless NIRS, which also provide the possibility to record not only relative changes in O2Hb but also tissue saturation index, which is an absolute measure of oxygenated hemoglobin. Another limitation of this study was that the sample size (*n* = 25) may not have been large enough to detect changes in the discharge rate and metabolic activity of TA muscle. This may have resulted in type II errors. However, the estimates from this study can be utilized to calculate the sample size for future trials evaluating the effect of spinal manipulation on metabolic characteristics. Lastly, as it is difficult to achieve adequate blinding when assessing manual interventions [78], the lack of blinding of participants and chiropractors may have added a bias.

## 5. Conclusions

In this group of relatively healthy participants, a single session of spinal manipulation resulted in increased plantar flexor muscle strength and muscle conduction velocity. Understanding how chiropractic care can have an impact on muscle strength does have implications for both healthy individuals and a variety of patient populations. This basic science study provides a better understanding of the mechanism behind the effects of chiropractic adjustments. However, the exact clinical implications this has for these various populations are yet to be identified through clinical trials. No change in the discharge rate and oxygen consumption was observed. The increase in conduction velocity could be explained by the recruitment of new units, though the mechanisms for the increase in maximum strength are still open for further research. Further research is also required to investigate the longer term and potential functional effects of spinal manipulation in a variety of patients who may benefit from improved muscle function and greater motor unit recruitment.

## References

[1] Jenkins H.J., Downie A.S., Moore C.S., French S.D. (2018). Current evidence for spinal X-ray use in the chiropractic profession: A narrative review. Chiropr. Man. Ther..

[2] McGill S.M. (1999). Stability: From biomechanical concept to chiropractic practice. J. Can. Chiropr. Assoc..

[3] Gross A., Langevin P., Burnie S.J., Bédard-Brochu M.S., Empey B., Dugas E., Faber-Dobrescu M., Andres C., Graham N., Goldsmith C.H. (2015). Manipulation and mobilisation for neck pain contrasted against an inactive control or another active treatment. Cochrane Database Syst. Rev..

[4] Gross A., Miller J., D’Sylva J., Burnie S.J., Goldsmith C.H., Graham N., Haines T., Brønfort G., Hoving J.L. (2010). Manipulation or mobilisation for neck pain: A Cochrane Review. Man. Ther..

[5] Bryans R., Decina P., Descarreaux M., Duranleau M., Marcoux H., Potter B., Ruegg R.P., Shaw L., Watkin R., White E. (2014). Evidence-based guidelines for the chiropractic treatment of adults with neck pain. J. Manip. Physiol. Ther..

[6] Goertz C.M., Pohlman K.A., Vining R.D., Brantingham J.W., Long C.R. (2012). Patient-centered outcomes of high-velocity, low-amplitude spinal manipulation for low back pain: A systematic review. J. Electromyogr. Kinesiol..

[7] Ruddock J.K., Sallis H., Ness A., Perry R.E. (2016). Spinal Manipulation vs. Sham Manipulation for Nonspecific Low Back Pain: A Systematic Review and Meta-analysis. J. Chiropr. Med..

[8] Bryans R., Descarreaux M., Duranleau M., Marcoux H., Potter B., Ruegg R., Shaw L., Watkin R., White E. (2011). Evidence-based guidelines for the chiropractic treatment of adults with headache. J. Manip. Physiol. Ther..

[9] Haavik H., Murphy B. (2012). The role of spinal manipulation in addressing disordered sensorimotor integration and altered motor control. J. Electromyogr. Kinesiol..

[10] Christiansen T.L., Niazi I.K., Holt K., Nedergaard R.W., Duehr J., Allen K., Marshall P., Türker K.S., Hartvigsen J., Haavik H. (2018). The effects of a single session of spinal manipulation on strength and cortical drive in athletes. Eur. J. Appl. Physiol..

[11] Holt K., Niazi I.K., Nedergaard R.W., Duehr J., Amjad I., Shafique M., Anwar M.N., Ndetan H., Turker K.S., Haavik H. (2019). The effects of a single session of chiropractic care on strength, cortical drive, and spinal excitability in stroke patients. Sci. Rep..

[12] Haavik H., Murphy B. (2011). Subclinical neck pain and the effects of cervical manipulation on elbow joint position sense. J. Manipulative Physiol. Ther..

[13] Holt K.R., Haavik H., Lee A.C.L., Murphy B., Elley C.R. (2016). Effectiveness of Chiropractic Care to Improve Sensorimotor Function Associated with Falls Risk in Older People: A Randomized Controlled Trial. J. Manipulative Physiol. Ther..

[14] (2017). The Rubicon Group Definition and Position Statement on the Chiropractic Subluxation. http://www.therubicongroup.org/#/policies/.

[15] Niazi I.K., Türker K.S., Flavel S., Kinget M., Duehr J., Haavik H. (2015). Changes in H-reflex and V-waves following spinal manipulation. Exp. Brain Res..

[16] Daligadu J., Haavik H., Yielder P.C., Baarbe J., Murphy B. (2013). Alterations in cortical and cerebellar motor processing in subclinical neck pain patients following spinal manipulation. J. Manipulative Physiol. Ther..

[17] Haavik H., Niazi I.K., Jochumsen M., Sherwin D., Flavel S., Türker K.S. (2017). Impact of spinal manipulation on cortical drive to upper and lower limb muscles. Brain Sci..

[18] Haavik H., Niazi I.K., Jochumsen M., Uginčius P., Sebik O., Yılmaz G., Navid M.S., Özyurt M.G., Türker K.S. (2018). Chiropractic spinal manipulation alters TMS induced I-wave excitability and shortens the cortical silent period. J. Electromyogr. Kinesiol..

[19] Haavik-Taylor H., Murphy B. (2007). Cervical spine manipulation alters sensorimotor integration: A somatosensory evoked potential study. Clin. Neurophysiol..

[20] Taylor H.H., Murphy B. (2008). Altered Sensorimotor Integration with Cervical Spine Manipulation. J. Manipulative Physiol. Ther..

[21] Taylor H.H., Murphy B. (2010). Altered Central Integration of Dual Somatosensory Input After Cervical Spine Manipulation. J. Manipulative Physiol. Ther..

[22] Taylor H.H., Murphy B. (2010). The Effects of Spinal Manipulation on Central Integration of Dual Somatosensory Input Observed After Motor Training: A Crossover Study. J. Manipulative Physiol. Ther..

[23] Lelic D., Niazi I.K., Holt K., Jochumsen M., Dremstrup K., Yielder P., Murphy B., Drewes A.M., Haavik H. (2016). Manipulation of dysfunctional spinal joints affects sensorimotor integration in the prefrontal cortex: A brain source localization study. Neural Plast..

[24] Navid M.S., Lelic D., Niazi I.K., Holt K., Mark E.B., Drewes A.M., Haavik H. (2019). The effects of chiropractic spinal manipulation on central processing of tonic pain—A pilot study using standardized low-resolution brain electromagnetic tomography (sLORETA). Sci. Rep..

[25] Haavik H., Murphy B.A. (2007). Transient modulation of intracortical inhibition following spinal manipulation. Chiropr. J. Aust..

[26] Allison T., McCarthy G., Wood C.C., Darcey T.M., Spencer D.D., Williamson P.D. (1989). Human cortical potentials evoked by stimulation of the median nerve. II. Cytoarchitectonic areas generating short-latency activity. J. Neurophysiol..

[27] Allison T., McCarthy G., Wood C.C., Williamson P.D., Spencer D.D. (1989). Human cortical potentials evoked by stimulation of the median nerve. II. Cytoarchitectonic areas generating long-latency activity. J. Neurophysiol..

[28] Waberski T.D., Buchner H., Perkuhn M., Gobbelé R., Wagner M., Kücker W., Silny J. (1999). N30 and the effect of explorative finger movements: A model of the contribution of the motor cortex to early somatosensory potentials. Clin. Neurophysiol..

[29] Kaňovský P., Bareš M., Rektor I. (2003). The selective gating of the N30 cortical component of the somatosensory evoked potentials of median nerve is different in the mesial and dorsolateral frontal cortex: Evidence from intracerebral recordings. Clin. Neurophysiol..

[30] Allison T., Mccarthy G., Wood C.C., Jones S.J. (1991). Potentials evoked in human and monkey cerebral cortex by stimulation of the median nerve: A review of scalp and intracranial recordings. Brain.

[31] Desmedt J.E., Cheron G. (1981). Non-cephalic reference recording of early somatosensory potentials to finger stimulation in adult or aging normal: Differentiation of widespread N18 and contralateral N20 from the prerolandic p22 and N30 components. Electroencephalogr. Clin. Neurophysiol..

[32] Desmedt J.E., Huy N.T., Bourguet M. (1983). The cognitive P40, N60 and P100 components of somatosensory evoked potentials and the earliest electrical signs of sensory processing in man. Electroencephalogr. Clin. Neurophysiol..

[33] Mauguière F., Desmedt J.E., Courjon J. (1983). Astereognosis and dissociated loss of frontal or parietal components of somatosensory evoked potentials in hemispheric lesions: Detailed correlations with clinical signs and computerized tomographic scanning. Brain.

[34] Rossini P.M., Babiloni F., Bernardi G., Cecchi L., Johnson P.B., Malentacca A., Stanzione P., Urbano A. (1989). Abnormalities of short-latency somatosensory evoked potentials in parkinsonian patients. Electroencephalogr. Clin. Neurophysiol. Evoked Potentials.

[35] Rossini P.M., Gigli G.L., Marciani M.G., Zarola F., Caramia M. (1987). Non-invasive evaluation of input-output characteristics of sensorimotor cerebral areas in healthy humans. Electroencephalogr. Clin. Neurophysiol. Evoked Potentials.

[36] Cheron G., Borenstein S. (1991). Gating of the early components of the frontal and parietal somatosensory evoked potentials in different sensory-motor interference modalities. Electroencephalogr. Clin. Neurophysiol. Evoked Potentials.

[37] Cheron G., Borenstein S. (1992). Mental movement simulation affects the N30 frontal component of the somatosensory evoked potential. Electroencephalogr. Clin. Neurophysiol. Evoked Potentials.

[38] Rossi S., Della Volpe R., Ginanneschi F., Ulivelli M., Bartalini S., Spidalieri R., Rossi A. (2003). Early somatosensory processing during tonic muscle pain in humans: Relation to loss of proprioception and motor “defensive” strategies. Clin. Neurophysiol..

[39] Duchateau J., Semmler J.G., Enoka R.M. (2006). Training adaptations in the behavior of human motor units. J. Appl. Physiol..

[40] Folland J.P., Williams A.G. (2007). The adaptations to strength training: Morphological and neurological contributions to increased strength. Sports Med..

[41] Kidgell D.J., Bonanno D.R., Frazer A.K., Howatson G., Pearce A.J. (2017). Corticospinal responses following strength training: A systematic review and meta-analysis. Eur. J. Neurosci..

[42] Narici M.V., Hoppeler H., Kayser B., Landoni L., Claassen H., Gavardi C., Conti M., Cerretelli P. (1996). Human quadriceps cross-sectional area, torque and neural activation during 6 months strength training. Acta Physiol. Scand..

[43] Gandevia S.C. (2001). Spinal and supraspinal factors in human muscle fatigue. Physiol. Rev..

[44] Vila-Chã C., Falla D., Correia M.V., Farina D. (2012). Adjustments in motor unit properties during fatiguing contractions after training. Med. Sci. Sports Exerc..

[45] Vila-Chã C., Falla D., Farina D. (2010). Motor unit behavior during submaximal contractions following six weeks of either endurance or strength training. J. Appl. Physiol..

[46] Farina D., Merletti R., Enoka R.M. (2004). The extraction of neural strategies from the surface EMG. J. Appl. Physiol..

[47] Methenitis S., Karandreas N., Spengos K., Zaras N., Stasinaki A.N., Terzis G. (2016). Muscle Fiber Conduction Velocity, Muscle Fiber Composition, and Power Performance. Med. Sci. Sports Exerc..

[48] Hargreaves M., Spriet L.L. (2020). Skeletal muscle energy metabolism during exercise. Nat. Metab..

[49] McCully K.K., Hamaoka T. (2000). Near-infrared spectroscopy: What can it tell us about oxygen saturation in skeletal muscle?. Exerc. Sport Sci. Rev..

[50] Hamaoka T., McCully K.K., Niwayama M., Chance B. (2011). The use of muscle near-infrared spectroscopy in sport, health and medical sciences: Recent developments. Philos. Trans. R. Soc. A Math. Phys. Eng. Sci..

[51] Grassi B., Quaresima V. (2016). Near-infrared spectroscopy and skeletal muscle oxidative function in vivo in health and disease: A review from an exercise physiology perspective. J. Biomed. Opt..

[52] Allen D.G., Lamb G.D., Westerblad H. (2008). Skeletal Muscle Fatigue: Cellular Mechanisms. Physiol. Rev..

[53] Enoka R.M., Duchateau J. (2008). Muscle fatigue: What, why and how it influences muscle function. J. Physiol..

[54] Hebert J.J., Stomski N.J., French S.D., Rubinstein S.M. (2015). Serious Adverse Events and Spinal Manipulative Therapy of the Low Back Region: A Systematic Review of Cases. J. Manipulative Physiol. Ther..

[55] Holt K., Russell D., Cooperstein R., Young M., Sherson M., Haavik H. (2018). Interexaminer reliability of a multidimensional battery of tests used to assess for vertebral subluxations. Chiropr. J. Aust..

[56] Masuda T., Miyano H., Sadoyama T. (1985). The Position of Innervation Zones in the Biceps Brachii Investigated by Surface Electromyography. IEEE Trans. Biomed. Eng..

[57] Kamavuako E.N., Scheme E.J., Englehart K.B. (2014). Combined surface and intramuscular EMG for improved real-time myoelectric control performance. Biomed. Signal Process. Control.

[58] Farina D., Pozzo M., Merlo E., Bottin A., Merletti R. (2004). Assessment of average muscle fiber conduction velocity from surface EMG signals during fatiguing dynamic contractions. IEEE Trans. Biomed. Eng..

[59] McGill K.C., Lateva Z.C., Marateb H.R. (2005). EMGLAB: An interactive EMG decomposition program. J. Neurosci. Methods.

[60] Ryan T.E., Erickson M.L., Brizendine J.T., Young H.-J., McCully K.K. (2012). Noninvasive evaluation of skeletal muscle mitochondrial capacity with near-infrared spectroscopy: Correcting for blood volume changes. J. Appl. Physiol..

[61] Thomas P.K., Sears T.A., Gilliatt R.W. (1959). The range of conduction velocity in normal motor nerve fibres to the small muscles of the hand and foot. J. Neurol. Neurosurg. Psychiatry.

[62] Hillermann B., Gomes A.N., Korporaal C., Jackson D. (2006). A pilot study comparing the effects of spinal manipulative therapy with those of extra-spinal manipulative therapy on quadriceps muscle strength. J. Manip. Physiol. Ther..

[63] Suter E., McMorland G., Herzog W., Bray R. (1999). Decrease in quadriceps inhibition after sacroiliac joint manipulation in patients with anterior knee pain. J. Manip. Physiol. Ther..

[64] Haavik H., Özyurt M.G., Niazi I.K., Holt K., Nedergaard R.W., Yilmaz G., Türker K.S. (2018). Chiropractic manipulation increases maximal bite force in healthy individuals. Brain Sci..

[65] Dunning J., Rushton A. (2009). The effects of cervical high-velocity low-amplitude thrust manipulation on resting electromyographic activity of the biceps brachii muscle. Man. Ther..

[66] Keller T.S., Colloca C.J. (2000). Mechanical force spinal manipulation increases trunk muscle strength assessed by electromyography: A comparative clinical trial. J. Manipulative Physiol. Ther..

[67] Vining R., Long C.R., Minkalis A., Gudavalli M.R., Xia T., Walter J., Coulter I., Goertz C.M. (2020). Effects of Chiropractic Care on Strength, Balance, and Endurance in Active-Duty U.S. Military Personnel with Low Back Pain: A Randomized Controlled Trial. J. Altern. Complement. Med..

[68] Duchateau J., Baudry S. (2014). Maximal discharge rate of motor units determines the maximal rate of force development during ballistic contractions in human. Front. Hum. Neurosci..

[69] Del Vecchio A., Casolo A., Negro F., Scorcelletti M., Bazzucchi I., Enoka R., Felici F., Farina D. (2019). The increase in muscle force after 4 weeks of strength training is mediated by adaptations in motor unit recruitment and rate coding. J. Physiol..

[70] Del Vecchio A., Negro F., Holobar A., Casolo A., Folland J.P., Felici F., Farina D. (2019). You are as fast as your motor neurons: Speed of recruitment and maximal discharge of motor neurons determine the maximal rate of force development in humans. J. Physiol..

[71] Blijham P.J., Ter Laak H.J., Schelhaas H.J., Van Engelen B.G.M., Stegeman D.F., Zwarts M.J. (2006). Relation between muscle fiber conduction velocity and fiber size in neuromuscular disorders. J. Appl. Physiol..

[72] Del Vecchio A., Negro F., Felici F., Farina D. (2017). Associations between motor unit action potential parameters and surface EMG features. J. Appl. Physiol..

[73] Fisher M.A. (1992). AAEM minimonograph #13: H reflexes and F waves: Physiology and clinical indications. Muscle Nerve.

[74] Tucker K.J., Tuncer M., Türker K.S. (2005). A review of the H-reflex and M-wave in the human triceps surae. Hum. Mov. Sci..

[75] Vila-Chã C., Falla D., Correia M.V., Farina D. (2012). Changes in H reflex and V wave following short-term endurance and strength training. J. Appl. Physiol..

[76] Aagaard P., Simonsen E.B., Andersen J.L., Magnusson P., Dyhre-Poulsen P. (2002). Neural adaptation to resistance training: Changes in evoked V-wave and H-reflex responses. J. Appl. Physiol..

[77] Berchicci M., Lucci G., Di Russo F. (2013). Benefits of physical exercise on the aging brain: The role of the prefrontal cortex. J. Gerontol. Ser. A Biol. Sci. Med. Sci..

[78] Chaibi A., Benth J.Š., Russell M.B. (2015). Validation of placebo in a manual therapy randomized controlled trial. Sci. Rep..

